# Understanding Health-Related Discussions on Reddit: Development of a Topic Assignment Method and Exploratory Analysis

**DOI:** 10.2196/55309

**Published:** 2025-01-29

**Authors:** Garrett J Chan, Mark Fung, Jill Warrington, Sarah A Nowak

**Affiliations:** 1 Departments of Pathology and Laboratory Medicine University of California, San Francisco San Francisco, CA United States; 2 Larner College of Medicine University of Vermont Burlington, VT United States

**Keywords:** digital health, internet, open data, social networking, social media

## Abstract

**Background:**

Social media has become a widely used way for people to share opinions about health care and medical topics. Social media data can be leveraged to understand patient concerns and provide insight into why patients may turn to the internet instead of the health care system for health advice.

**Objective:**

This study aimed to develop a method to investigate Reddit posts discussing health-related conditions. Our goal was to characterize these topics and identify trends in these social media–based medical discussions.

**Methods:**

Using an initial query, we collected 1 year of Reddit posts containing the phrases “get tested” and “get checked.” These posts were manually reviewed, and subreddits containing irrelevant posts were excluded from analysis. This selection of posts was manually read by the investigators to categorize posts into topics. A script was developed to automatically assign topics to additional posts based on keywords. Topic and keyword selections were refined based on manual review for more accurate topic assignment. Topic assignment was then performed on the entire 1-year Reddit dataset containing 347,130 posts. Related topics were grouped into broader medical disciplines. Analysis of the topic assignments was then conducted to assess condition and medical topic frequencies in medical condition–focused subreddits and general subreddits.

**Results:**

We created an automated algorithm to assign medical topics to Reddit posts. By iterating through multiple rounds of topic assignment, we improved the accuracy of the algorithm. Ultimately, this algorithm created 82 topics sorted into 17 broader medical disciplines. Of all topics, sexually transmitted infections (STIs), eye disorders, anxiety, and pregnancy had the highest post frequency overall. STIs comprised 7.44% (5876/78,980) of posts, and anxiety comprised 5.43% (4289/78,980) of posts. A total of 34% (28/82) of the topics comprised 80% (63,184/78,980) of all posts. Of the medical disciplines, those with the most posts were psychiatry and mental health; genitourinary and reproductive health; infectious diseases; and endocrinology, nutrition, and metabolism. Psychiatry and mental health comprised 26.6% (21,009/78,980) of posts, and genitourinary and reproductive health comprised 13.6% (10,741/78,980) of posts. Overall, most posts were also classified under these 4 medical disciplines. During analysis, subreddits were also classified as *general* if they did not focus on a specific health issue and *topic-specific* if they discussed a specific medical issue. Topics that appeared most frequently in the top 5 in general subreddits included addiction and drug anxiety, attention-deficit/hyperactivity disorder, abuse, and STIs. In topic-specific subreddits, most posts were found to discuss the topic of that subreddit.

**Conclusions:**

Certain health topics and medical disciplines are predominant on Reddit. These include topics such as STIs, eye disorders, anxiety, and pregnancy. Most posts were classified under the medical disciplines of psychiatry and mental health, as well as genitourinary and reproductive health.

## Introduction

### Background

With over 3.8 billion people regularly sharing information about their lives on social media platforms [1], social media has become a ubiquitous part of daily life. In addition to building relationships with other people, social media helps users find information on disciplines that they are concerned about or interested in. Social media platforms have become a commonly used way to discuss problems and seek advice [2-4].

Given its prevalent use, social media content may provide insight into individual attitudes and opinions about health. The anonymous nature of certain social media platforms allows users to choose to discuss potentially embarrassing subjects without an expectation of privacy. Deidentified information can be used for research purposes to understand social behavior as it is impossible to associate a specific comment with a single person.

Analysis of social media content has other strengths compared to more traditional methods such as surveys. For example, surveys may be influenced by social desirability bias, and respondents may not be as open when discussing sensitive or potentially embarrassing conditions [5] such as drug use or sexually transmitted infections (STIs). Several studies have demonstrated that internet-based platforms can circumvent or mitigate this limitation. For example, as Google search history reflects an active process (seeking information) rather than stated beliefs, it may be more accurate than surveys for some stigmatized beliefs in particular [6]. People have a variety of motivations for using social media, including information seeking, information sharing, and expression of opinion [2]. While previous work has also shown that online content becomes more shared, or viral, when it invokes strong, activating emotions, either positive or negative [7], some studies have suggested that individuals may be more honest with sharing their thoughts online.

An advantage of the use of social media to learn about people’s behaviors and sentiments is that we can obtain large volumes of data in a short period and user-generated content can be processed quickly [8,9]. Social media websites count among the most trafficked ones in the United States. Facebook is the 6th most popular website in the United States at the time of writing, Reddit is the 7th, Instagram is the 18th, and Twitter (subsequently rebranded X) is the 33rd. The variety of topics discussed on social media is wide-ranging, and these topics include information about people’s health concerns as users can pose and answer questions about health-related conditions on internet forums.

Reddit has over 270 million monthly users [10,11] who can post first-person accounts of illness and interactions with the health care system and leave comments on other users’ posts about their experiences with similar illnesses. At the time of writing, Reddit ranks 17th in global engagement [12]. In addition, 42.8% of Reddit site traffic comes from search engines rather than direct visits to the site. For comparison, only 12.4% of Twitter traffic comes from searches, which may mean that a greater proportion of visits to Reddit comes from internet users doing a general search for information rather than choosing to go to a specific website. Therefore, many internet users reach Reddit from search engines, making the site an important source of information to both Reddit users and nonusers.

While the spectrum of discussion about health on social media is broad, studies of sites such as Reddit, Twitter, and Facebook have been able to examine disease- and health concern–specific discussions. This includes detection of opioid addiction based on Twitter posts [13] and discussion of colorectal cancer, breast cancer, and diabetes through analysis of Twitter posts and Facebook groups [14]. One study also categorized user data from Twitter posts into 4 specific disease-related topics: *flu*, *depression*, *pregnancy*, and *eating disorders* [15]. Other studies on Reddit have sought to detect depression [16], detect suicidality among opioid users [17], and characterize weight loss discussions [18]. These works have demonstrated the ability to select specific topics from social media data and draw conclusions about how these topics are encountered in the social media sphere. Multiple studies have characterized language markers to evaluate discussions on medical topics, giving each medical topic a unique pattern of markers [19]. In all, each medical topic can be thought of as having a social media “gene” that conceptualizes it as having a recognizable identity on social media that can be tracked and analyzed [20].

The specific system architecture and features of different social media platforms make each more optimally suited for different types of studies (Table 1). For example, differences in information organization, post length, and rules for content creation can influence the analysis. The use of Reddit, for example, is well suited for evaluation of stigmatized topics and topic-based conversations. On Reddit, posts are organized into groups by topic, and users can interact through specific forums, called subreddits, to find discussions related to the subject they are interested in. A post’s visibility in a subreddit is determined by user upvotes and downvotes. In addition, posts may be removed by moderators if they violate subreddit-specific community rules [21]. Therefore, subreddits are a dedicated space for people to address a topic with the expectation of pertinent discussion. Previous work has noted that the topic-based organization of Reddit content is appropriate for studying specific health topics in greater detail [22]. Furthermore, some subreddits are question and answer–based. On the subreddit *r/askdocs*, users can seek answers about medical topics from health professionals who have been verified by subreddit moderators. While these subreddits do not coalesce around a certain topic, they allow users to ask questions to content experts anonymously, a function difficult to find on other social media platforms.

**Table 1:** Comparison of select social media platforms by content, organization, level of anonymity, moderation, and number of articles on PubMed.

	Reddit	Twitter	Facebook
Content length	40,000-character limit	280-character limit	No limit
Content organization and sharing	Users search for posts of interest; posts organized in topic-based subreddits	On the basis of accounts that a user follows; users can search for common themes using hashtags	Often based on real-life connections
Anonymity	May be completely anonymous	May or may not be anonymous	Real-name culture encouraged
Moderation	User moderated; subreddits have content guidelines	Platform moderated	Platform moderated
PubMed articles, N	277	5769	5030

Despite discussion forums such as Reddit having features that distinguish them from platforms such as Twitter and Facebook in important ways, comparatively little research has been conducted investigating health beliefs and discussions on Reddit. A number of text analysis studies on social media platforms have been conducted—at the time of writing, there are 5769 articles on PubMed discussing Twitter and 5030 about Facebook. However, only 277 articles have been published discussing Reddit [23]. For an internet user searching for information on a specific subject, Reddit represents a useful starting point because of its topic-focused organization.

### Objectives

We report a text analysis of health concerns and topics reflected in users’ Reddit posts during 2019. Given the previous studies on using social media to study health and medicine, we wanted to continue this work while looking at medical topics on another platform. In addition to the relative lack of study compared to other social media platforms, we chose to investigate Reddit because it has features that differentiate it from heretofore more frequently studied social media platforms: specific, topic-based discussions; greater anonymity; and user moderators. We wanted to capture posts from a variety of health-relevant subreddits discussing seeking or receiving testing and diagnostic advice.

## Methods

### Ethical Considerations

The RAND Corporation was consulted regarding institutional review board approval. The RAND Human Subject Protection Committee (the RAND Institutional Review Board) determined that the project did not involve human participants as defined by the regulations of Title 45 of the Code of Federal Regulations Part 46.102(f) and, therefore, was not subject to further review. We did not explicitly obtain a waiver of informed consent; however, the determination of the absence of human participants implies a waiver of the need for documented informed consent. In addition, all data were anonymous, and obtaining informed consent would require identifying individuals, which would also be a violation of the Reddit terms of service.

### Overview

**Figure 1:**
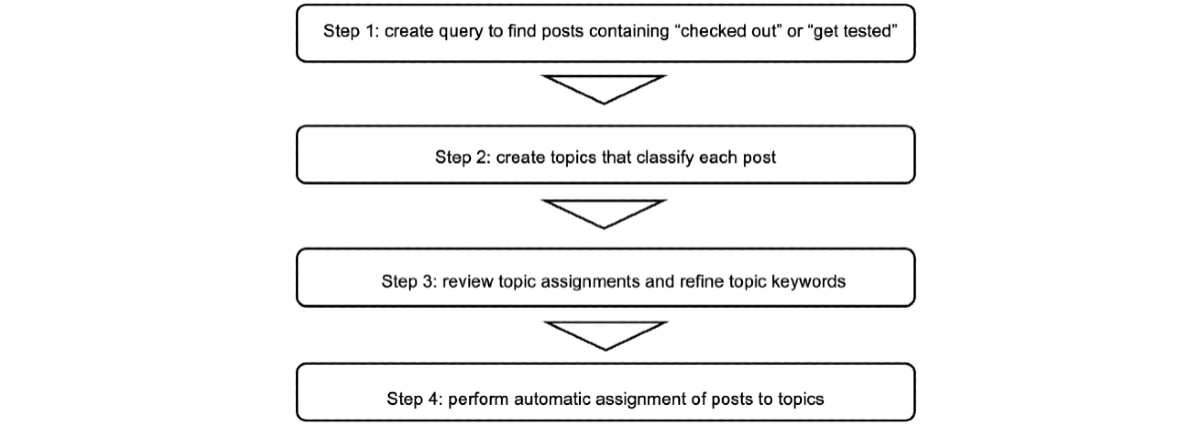
Overview of analysis steps—the overall methodology for gathering posts, creating topics, and automatically assigning posts to topics.

**Figure 2:**
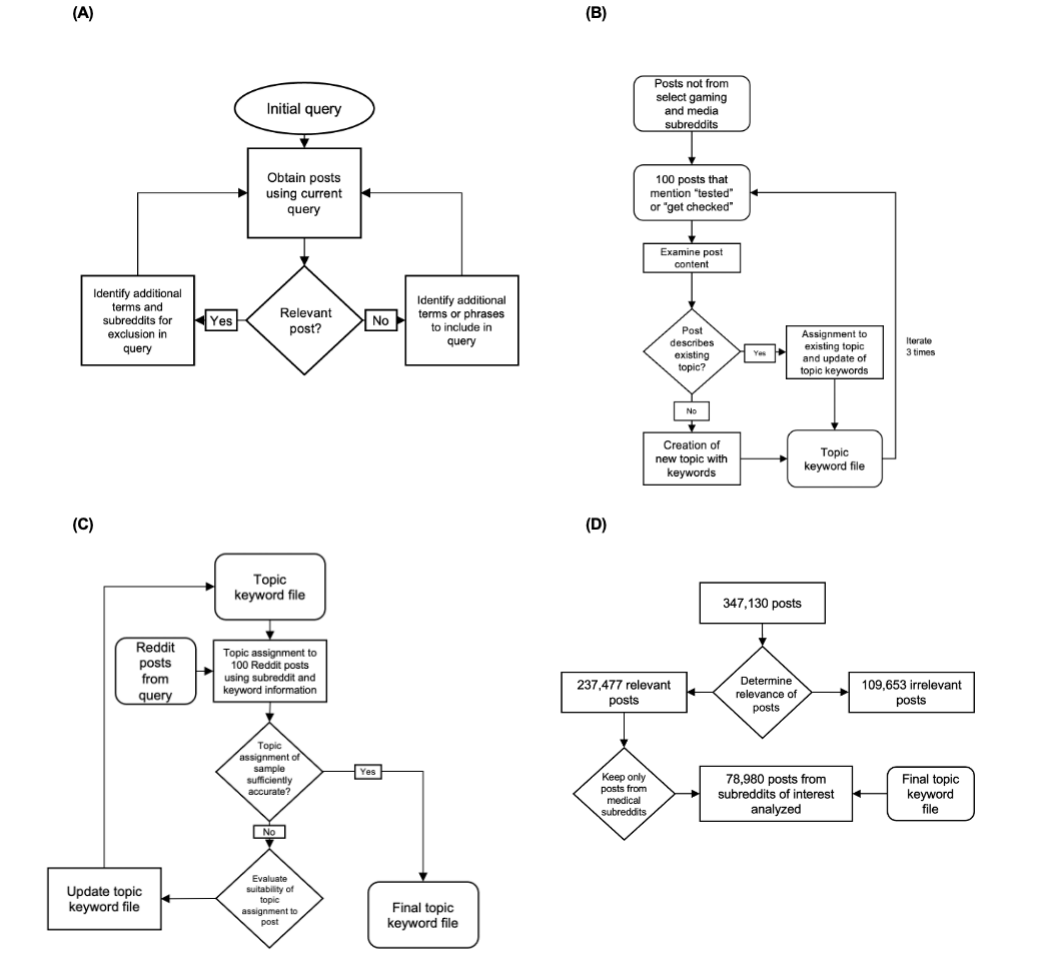
Detailed flow diagrams of analysis steps 1 to 4. (A) Step 1: selection of Reddit posts. Posts from January 1, 2019, to December 31, 2019, were considered in this study. (B) Step 2: identification of topic keywords. Topics were manually created from three 100-post samples, and keywords were assigned to each topic. (C) Step 3: topic assignment. Topics were manually assigned to additional 100-post samples based on the topic keyword file. (D) Step 4: automatic assignment of posts for analysis. The algorithm using the final topic keyword file was run on 78,980 posts chosen from the overall corpus of 347,130 posts.

We conducted our analysis in 4 steps (Figure 1) designed to balance false positives and false negatives. In step 1, we developed and refined a query to obtain posts about medical testing and advice about seeking diagnoses. In step 2, topics were created to classify the posts. Subreddits that clearly contained irrelevant posts were identified and excluded to improve the efficiency of the topic creation process. In step 3, posts assigned to the developed topics were reviewed, and the keywords used for topic assignment were refined. The process for identifying subreddits with relevant posts to be included in the analysis was refined as well. In step 4, keywords were used to do a final automatic assignment to a set of posts from a 12-month period. The 4 steps are shown in more detail in Figure 2 and are described in the following sections.

### Step 1: Query Creation and Refinement

First, we captured posts from Reddit that were relevant to testing and diagnosis. We developed a query in Crimson Hexagon’s Monitor application programming interface to obtain posts that contained variations of the phrases “getting tested” or “getting checked out” and excluded posts from subreddits that would be unlikely to be relevant to medicine or health care (Figure 2A and Table S1 in Multimedia Appendix 1). The query run was as follows: “((get OR got) AND ((test AND tested)OR(check OR checked)) AND for)~4” (Figure 2A). Posts gathered using this query included either the words “get” or “got” in conjunction with “test,” “tested,” “check,” or “checked” appearing within 4 words of each other. The posts collected were created between January 1, 2019, and December 31, 2019, and included the text content of the post, its title, and its date. A *post* could be the body of text created by the original poster or a comment from a user on an original post. In both cases, the content of a post during our analysis included the title of the original post to provide additional context.

To limit our false-positive rate, we first excluded subreddits tabulated on *r/listofsubreddits* (accessed November 18, 2019) that would be most irrelevant to health-related discussions (Table S1 in Multimedia Appendix 1). Posts not from these subreddits were included in the topic creation step.

### Step 2: Topic Creation

In this step, we manually read through 100 posts and labeled each with a de novo topic (Figure 2B). Topic creation was guided by the existing taxonomy in the *International Classification of Diseases, 10th Revision* (*ICD-10*) [24]. Topics were sorted into medical disciplines to optimize the analysis by clustering topics into larger groups.

These disciplines corresponded to chapter titles in the *ICD-10*. We then designated keywords that corresponded to each of these topics. For example, for the topic of STIs, keywords included “antibiotics,” “gonorrhea,” “chlamydia,” and “bacterial vaginosis.” Additional keywords were chosen from the corresponding article topic on UpToDate (Wolters Kluwer N.V.) [25], a reference for health care providers covering the diagnosis, pathophysiology, and treatment of a broad spectrum of medical conditions. In addition, several topics related to the health care system but not to a disease or topic were created, such as medical billing. Finally, for posts that did not fit with any of the aforementioned topics, a *nonmedical* topic was devised. Posts that corresponded to an existing topic were assigned to that topic.

The topics and disciplines that we formulated corresponded to the diagnoses and chapters in the *ICD-10*. A list of topics with their corresponding category is available in Table S2 in Multimedia Appendix 1. This categorization system is similar to the categorization of diseases and topics that health care providers follow. Therefore, our topics and medical disciplines were validated by an existing and accepted hierarchy of disease classification. We chose to use the *ICD-10* over other controlled vocabulary systems such as the Medical Subject Headings thesaurus of the National Library of Medicine and the Logical Observation Identifiers Names and Codes database because the *ICD-10* is the system that most physicians are in contact with and is the most targeted at covering the diagnosis of diseases. In contrast, the Medical Subject Headings thesaurus is a broader system for indexing publications that encompasses anatomy, organisms, and drugs, among many other topics.

During this process, additional irrelevant subreddits and terms were excluded from the query. In total, 3 iterations of keyword selection, each performed on a 100-post set, were completed. This generated our initial topic keyword file.

### Step 3: Topic Refinement and Determination of False-Positive Rate as Means of Validating Topic Assignment

To begin automatically assigning topics to posts (Figure 2C), we created a script in R (R Foundation for Statistical Computing) that first put each post into tidy format using the *tidyverse* package [26]. The script then assigned a single topic to each post based on words found in the post and on the keyword file. With further manual analyses of more posts, we added more keywords to each topic with the goal of improving future topic assignment in later iterations.

We read through 100-post samples to add additional keywords to topics. After each run, we manually validated the accuracy of the topic assignments and iterated through this review process for 15 runs. Our topic assignments were reviewed by SAN and GC. Keyword classifications were reviewed by SAN and GC, as well as by MF and JW, 2 clinical pathologists. Accuracy was determined by reading the title and content of the posts and determining whether the topic captured the essence of the post. Topic assignment was designated as either correct or incorrect. We maintained objectivity by blinding the topic reviewer to each post’s topic as assigned by the script before manually assigning a topic to that post. An example of a post and its assigned topic is as follows:

POST: I find that when I go through bouts of anxiety the mornings are usually the hardest, no appetite, tired etc, but as the day goes on I find I feel better, even to the extent by evening time I feel “normal.”

ASSIGNED TOPIC: Anxiety

We then determined how many posts’ topics had been correctly assigned per run and calculated an accuracy rate. During this process, we improved our script by allowing multi-word keywords and lemmatizing keywords. In addition, more subreddits were excluded to improve accuracy (Table S3 in Multimedia Appendix 1).

In addition, our algorithm excluded irrelevant posts based on irrelevant terms and subreddits determined in step 2. During the manual review process, we reviewed which posts were determined to be irrelevant by the algorithm to determine the accuracy and efficacy of using those exclusion methods [27-29]. Then, in each iteration, we calculated the false-positive rate, defined as the number of relevant posts divided by the total number of posts subtracted from 1 [30].

Posts were defined as relevant to health care if the main subject of the post related to seeking a diagnosis for a medical condition, discussion of treatment and management of a medical condition, or medical billing. An example of such a post is as follows:

Licensed skincare practitioner here. Get it checked. SKs [seborrheic keratoses] can itch and flake. Also may be BCC.

An example of an irrelevant post is as follows:

Him going around checking if his wife cleaned properly is not something that should be rationalised....

### Step 4: Analysis of Reddit Data From January 1, 2019, to December 31, 2019, and Subreddit Analysis

To restrict our final analysis to posts mostly likely to be relevant to health-related discussion, we further limited the posts in the final analysis to those from certain subreddits. We specifically selected the 77 health-related subreddits posted on *r/listofsubreddits* and manually read through these subreddits to check for relevance to health discussions. In addition, we chose 10 subreddits with the most posts from our data sample to gather a broad range of Reddit discussion and another 8 health-related subreddits not posted on *r/listofsubreddits* that we determined contained relevant posts. In total, we obtained 95 subreddits.

We used the topic keyword file to automatically assign topics and medical disciplines to the set of Reddit posts that matched the query described previously (Figure 2D). Of note, we found that only posts from 89% (85/95) of the subreddits were assigned to a topic, indicating that the other 10 subreddits did not contain any health-related discussion as determined by the algorithm. We manually read through 300 posts to determine the accuracy rate. The topic assignments then underwent further analysis to determine the distribution of topics and medical disciplines, distribution of posts in subreddits, and distribution of topics in each subreddit. Finally, we selected quotes from our quantitative results to provide human-relatable insights that illustrated what was discussed in each discipline, as previously done in other social media–mining research [27,31,32].

## Results

### Step 1: Determination of Relevant Posts, Subreddit Limitation, and Query Refinement

In total, 347,130 posts from January 1, 2019, to December 31, 2019, were downloaded from Reddit. Of those 347,130 posts, 109,653 (31.59%) belonged to subreddits that were determined to be irrelevant, and 237,477 (68.41%) came from relevant subreddits. Irrelevant subreddits were found to be those pertaining to games, popular media, and technology (Table S1 in Multimedia Appendix 1).

In step 1 (Figure 2A), the exclusion of irrelevant subreddits from our query decreased the false-positive rate of irrelevant posts from 59% to 29% (calculated from a random sample of 100 posts). We did not attempt to further decrease the false-positive rate in this step to avoid overexcluding subreddits and posts in the manual review step.

### Steps 2 and 3: Topic Creation and Validation

A total of 82 topics were created through the inductive workflow outlined previously and sorted into 17 medical disciplines. Each topic was assigned to only 1 medical discipline. We validated our choices of topics and medical disciplines by comparing these categorizations to the diseases and medical topics delineated in the *ICD-10*. Of the 82 topics we created, 80 (98%) had a corresponding topic in the *ICD-10*; the exceptions were the *medical billing* and *nonmedical* topics. The accuracy of the topic assignments in this step was 56.3% (SD 4.92%; SEM 4.62%).

### Step 4: Topics and Medical Disciplines

In step 4, subreddits to include in the final analysis were identified. While our objective in step 1 was to reduce the false-positive rate without being too restrictive (eliminating only subreddits least likely to contain medical discussion), our objective in this step was to keep only subreddits most likely to contain medical discussions. In total, 95 subreddits were chosen. These subreddits contained 78,980 posts.

Limiting posts to only relevant subreddits further decreased the false-positive rate from 29% to 15.5%. The accuracy rate in this step was 62.5% (SD 1.59%; SEM 0.793%), which was higher than in steps 1 and 3. Therefore, limiting posts to mainly medically relevant subreddits improved the relevance and accuracy compared to only excluding irrelevant subreddits.

**Figure 3:**
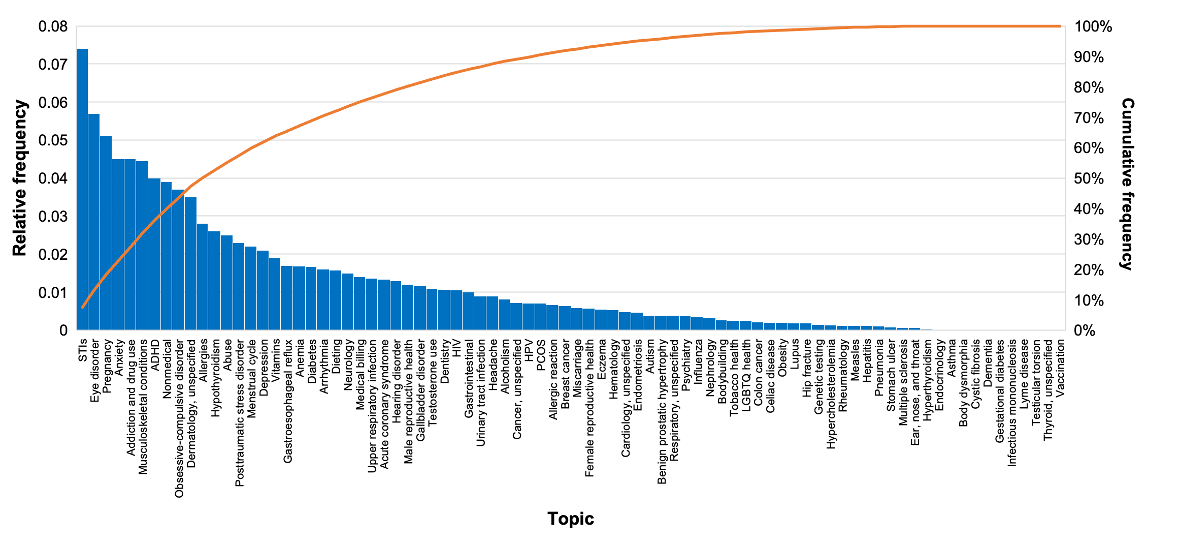
Relative frequency of all topics in descending order as determined by the topic assignment algorithm. The cumulative frequency of all topics is also shown. ADHD: attention-deficit/hyperactivity disorder; HPV: human papillomavirus; LGBTQ: lesbian, gay, bisexual, transgender, and queer; PCOS: polycystic ovary syndrome; PTSD: posttraumatic stress disorder; STI: sexually transmitted infection.

The relative frequency of each topic was calculated (Figure 3). STIs, eye disorders, anxiety, and pregnancy had the highest frequency at 7.44% (5876/78,980), 5.6% (4423/78,980), 5.43% (4289/78,980), and 5.09% (4020/78,890) of all posts, respectively. Interestingly, 10 topics had a 0% relative frequency: asthma, body dysmorphic disorder, cystic fibrosis, dementia, gestational diabetes, infectious mononucleosis, Lyme disease, testicular cancer, thyroid (unspecified), and vaccination. Nonmedical posts made up 3.87% (3057/78,890) of all posts. If including the *nonmedical* topic, 80% (63,184/78,890) of posts were categorized within the first 34% (28/82) of topics. The mean relative frequency of all topics was 1.22% (SD 0.15%).

As 34% (28/82) of the topics comprised 80% (63,184/78,890) of posts, this points to a skewed distribution of topics. In total, 61% (17/28) of those topics belonged to the top 4 medical disciplines (psychiatry and mental health; genitourinary and reproductive health; infectious diseases; and endocrinology, nutrition, and metabolism).

Topics were grouped into 17 broader medical disciplines (Table 2) based on the categorization scheme of the chapter codes in the *ICD-10*, as described previously. The most discussed disciplines were psychiatry and mental health; genitourinary (related to the urinary and reproductive systems) and reproductive health; infectious diseases; and endocrinology, nutrition, and metabolism, whose posts, respectively, constituted 26.6% (21,009/78,980), 13.6% (10,741/78,980), 8.95% (7069/78,980), and 8.91% (7037/78,980) of all analyzed posts. Psychiatry and mental health comprised most posts. Interestingly, most of the other medical disciplines were discussed with similar relative frequencies. The next most common medical discipline was hematology and immunology with a 5.88% (4644/78,890) relative frequency. The medical discipline with the lowest relative frequency was otolaryngology (related to medicine dealing with the ear, nose, and throat) at 0.57% (450/78,890).

**Table 2:** The relative frequency of the broader medical disciplines, into which the medical topics were sorted, shown in descending order.

Medical discipline	Relative frequency	
Psychiatry and mental health	0.266	
Genitourinary and reproductive health	0.136	
Infectious diseases	0.0895	
Endocrinology, nutrition, and metabolism	0.0891	
Hematology and immunology	0.0588	
Ophthalmology	0.0572	
Musculoskeletal and connective tissue conditions	0.0476	
Gastroenterology	0.0426	
Dermatology	0.0410	
Nonmedical	0.0387	
Neurology	0.0373	
Cardiovascular	0.0353	
Pulmonology	0.0183	
Medical; other	0.0160	
Neoplasms	0.0157	
Dentistry	0.0095	
Otolaryngology	0.0022	

The most common topics in the 4 most discussed medical disciplines are shown in Table 3. Within the medical discipline of psychiatry and mental health, the 3 most common topics were anxiety, addiction and drug use, and attention-deficit/hyperactivity disorder (ADHD) with relative frequencies of 20.42% (4289/21,009), 17.3% (3635/21,009), and 15.1% (3172/21,009), respectively. In the medical discipline of infectious diseases, the most common topic by a significant margin was STIs, making up 83.12% (5876/7069) of infectious disease posts. HIV and influenza made up 11.01% (778/7069) and 3.3% (233/7069) of posts, respectively. In genitourinary and reproductive health, pregnancy was the most common topic, comprising 37.43% (4020/10,741) of genitourinary and reproductive health posts, followed by menstrual cycle (1665/10,741, 15.5%) and male reproductive health (591/10,741, 5.5%). Finally, posts within the medical discipline of endocrinology, nutrition, and metabolism were more evenly distributed across topics. Hypothyroidism had the greatest number of posts (2013/7037, 28.61%), followed by vitamins (1534/7037, 21.8%) and diabetes (1316/7037, 18.7%).

**Table 3:** Relative frequency of topics on Reddit, as determined by our algorithm, within medical disciplines. The breakdown of the relative frequency of topics in the 4 most prevalent medical disciplines is as follows: psychiatry and mental health; genitourinary and reproductive health; infectious diseases; and endocrinology, nutrition, and metabolism. The medical disciplines are also shown in descending order by prevalence.

Medical discipline and topic		Relative frequency
**Psychiatry and mental health**		
	Anxiety	0.203
	Addiction and drug use	0.169
	ADHD^a^	0.151
	Obsessive-compulsive disorder	0.139
	Abuse	0.094
	PTSD^b^	0.087
	Depression	0.079
	Alcoholism	0.031
	Autism	0.010
	Psychiatry	0.0090
	Tobacco use	0.0085
	LGBTQ^c^ health	0.0051
	Body dysmorphic disorder	0.0015
	Dementia	0.0013
**Genitourinary and reproductive health**		
	Pregnancy	0.376
	Menstrual cycle	0.160
	Male reproductive health	0.088
	Testosterone use	0.081
	Urinary tract infection	0.068
	HPV^d^	0.053
	Miscarriage	0.044
	Female reproductive health	0.042
	Endometriosis	0.035
	Benign prostatic hypertrophy	0.028
	Nephrology; unspecified	0.024
	Gestational diabetes	0
	Testicular torsion	0
**Infectious diseases**		
	STIs^e^	0.831
	HIV	0.118
	Influenza	0.039
	Measles	0.013
	Infectious mononucleosis	0
	Lyme disease	0
	Vaccination	0
**Endocrinology, nutrition, and metabolism**		
	Hypothyroidism	0.286
	Vitamins	0.216
	Diabetes	0.188
	Dieting	0.178
	PCOS^f^	0.078
	Bodybuilding	0.029
	Obesity	0.022
	Hyperthyroidism	0.002
	Endocrinology	0.001
	Thyroid; unspecified	0

^a^ADHD: attention-deficit/hyperactivity disorder.

^b^PTSD: posttraumatic stress disorder.

^c^LGBTQ: lesbian, gay, bisexual, transgender, and queer.

^d^HPV: human papillomavirus.

^e^STI: sexually transmitted infection.

^f^PCOS: polycystic ovary syndrome.

### Posts per Subreddit

Of the 95 subreddits identified in step 4, a total of 10 (11%) did not contain any posts that were assigned to our topics and were excluded, leaving 85 final subreddits. These 85 subreddits contained 78,980 posts (Table 4). The subreddits with the most posts were those that we describe as *general*, or non–topic-specific, subreddits. The top 10 subreddits with the most posts included 6 general subreddits: *r/askreddit*, *r/askdocs*, *r/amitheasshole*, *r/medical*, *r/legaladvice*, and *r/advice*. The most popular subreddit was *r/askreddit* with 26.7% (21,086/78,980) of posts, followed by *r/askdocs* with 12.02% (9496/78,980) of posts. A total of 61.93% (48,912/78,890) of the posts analyzed were found in the top 5 subreddits by number of posts, and 79.91% (63,112/78,890) were found in the top 15 subreddits.

**Table 4:** Subreddits by number of posts analyzed showing the relative differences in the number of posts in each subreddit (N=78,980).

Subreddit	Posts analyzed, n (%)	
r/askreddit	21,086 (26.7)	
r/askdocs	9496 (12.02)	
r/amitheasshole	5473 (6.93)	
r/relationship_advice	5322 (6.74)	
r/relationships	2775 (3.51)	
r/medical	2495 (3.16)	
r/skincareaddiction	2268 (2.87)	
r/legaladvice	2227 (2.82)	
r/advice	2150 (2.72)	
r/teenagers	1974 (2.5)	
r/ADHD	1876 (2.38)	
r/babybumps	1775 (2.25)	
r/Testosterone	1746 (2.21)	
r/STD	1345 (1.7)	
r/Anxiety	1215 (1.54)	
r/Depression	1193 (1.51)	
r/pregnant	1166 (1.48)	
r/birthcontrol	1102 (1.4)	
r/childfree	966 (1.22)	
r/Hypothyroidism	880 (1.11)	
r/fitness	778 (0.99)	
r/MentalHealth	741 (0.94)	
r/Diabetes	699 (0.89)	
r/Dentistry	602 (0.76)	
r/science	489 (0.62)	
r/diagnoseme	467 (0.59)	
r/Infertility	446 (0.56)	
r/SuicideWatch	429 (0.54)	
r/Optometry	410 (0.52)	
r/Ibs	391 (0.5)	
r/CrohnsDisease	376 (0.48)	
r/Cancer	349 (0.44)	
r/Fibromyalgia	318 (0.4)	
r/ChronicPain	300 (0.38)	
r/MultipleSclerosis	279 (0.35)	
r/Aspergers	272 (0.34)	
r/bpd	235 (0.3)	
r/SleepApnea	230 (0.29)	
r/BipolarReddit	201 (0.25)	
r/cfs	188 (0.24)	
r/askdoctorsmeeee	175 (0.22)	
r/askdoctors	150 (0.19)	
r/Rheumatoid	127 (0.16)	
r/medicine	125 (0.16)	
r/Eczema	120 (0.15)	
r/Asthma	118 (0.15)	
r/InterstitialCystitis	117 (0.15)	
r/Nutrition	115 (0.15)	
r/Dementia	86 (0.11)	
r/askscience	84 (0.11)	
r/Healthcare	80 (0.1)	
r/StopSmoking	79 (0.1)	
r/Dysautonomia	77 (0.1)	
r/Psoriasis	75 (0.09)	
r/Keratoconus	72 (0.09)	
r/Pharmacy	59 (0.07)	
r/Vegetarian	52 (0.07)	
r/Massage	48 (0.06)	
r/KidneyStones	47 (0.06)	
r/GravesDisease	40 (0.05)	
r/Menieres	32 (0.04)	
r/Pilonidalcyst	31 (0.04)	
r/Posture	30 (0.04)	
r/Paleo	27 (0.03)	
r/Gastroparesis	24 (0.03)	
r/Hemophilia	22 (0.03)	
r/Psychology	17 (0.02)	
r/TroubledTeens	16 (0.02)	
r/PancreaticCancer	13 (0.02)	
r/Amblyopia	11 (0.01)	
r/LactoseIntolerant	9 (0.01)	
r/AlternativeHealth	8 (0.01)	
r/NaturalBeauty	7 (0.01)	
r/PublicHealth	6 (0.01)	
r/Transplant	5 (0.01)	
r/CareGiverSupport	3 (<0.01)	
r/hepc	3 (<0.01)	
r/chd	2 (<0.01)	
r/Flu	2 (<0.01)	
r/Juicing	1 (<0.01)	
r/Pescetarian	1 (<0.01)	
r/UKHealthcare	1 (<0.01)	
r/Dystonia	1 (<0.01)	
r/ItsNeverLupus	1 (<0.01)	
r/MacularDegeneration	1 (<0.01)	

### General Subreddits Versus Topic-Specific Subreddits

Subreddits that we defined as *general* were those that did not focus on a specific health topic. These 15 subreddits were *r/askreddit*, *r/askdocs*, *r/amitheasshole*, *r/relationship_advice*, *r_relationships*, *r/medical*, *r/legaladvice*, *r/advice*, *r/teenagers*, *r/fitness*, *r/science*, *r/diagnoseme*, *r/askdoctorsmeeee*, *r/askdoctors*, and *r/medicine*. Compared to topic-specific subreddits, a wider variety of topics was represented in these subreddits, and no one topic dominated over other topics in each subreddit. We identified the top 5 topics by number of posts in each subreddit. Among the general subreddits, these topics comprised, on average, 41.1% of posts (SD 12.9%; 95% CI 28.2%-54%). For comparison, among topic-specific subreddits, the top 5 topics on average comprised 70.3% of posts (SD 38.3%; 95% CI 32%-100%).

In 67% (10/15) of the general subreddits, STIs was one of the top 5 topics (Table 5). Notably, the second most prevalent topic in *r/science* was human papillomavirus, whereas the most prevalent topic in *r/healthcare* (not one of the top 15 general subreddits) was medical billing. Interestingly, we also noted that the top 5 topics in *r/askreddit*, which is not specifically targeted toward health issues, were musculoskeletal conditions, eye disorders, ADHD, addiction and drug use, and allergies. For comparison, in *r/askdocs*, the most prevalent topics were STIs, dermatology, musculoskeletal conditions, allergies, and anxiety. Mental health topics were common in the general subreddits. Anxiety appeared as a top-5 topic in 53% (8/15) of the general subreddits. In those 8 subreddits, an average of 6.27% (SD 0.017%) of all posts were assigned to the topic of anxiety. Addiction and drug use appeared as a top-5 topic in 33% (5/15) of the general subreddits, and an average of 10.9% (SD 5.30%) of all posts in those 5 subreddits were assigned to that topic. On the other hand, we found that topic-specific subreddits often had the topic of the same name as the most assigned topic (eg, the topic of ADHD would be the most assigned topic in the subreddit *r/ADHD*; Table 6).

**Table 5:** The top 5 topics by number of posts in the top 15 general subreddits (subreddits without a specific health-related topic). A description of the subreddit and the total number of posts in the subreddit are provided (N=78,980).

Subreddit	Description	Posts, n (%)	Top 5 topics in subreddit
r/askreddit	Open question-and-answer forum	21,086 (26.7)	Musculoskeletal conditions (n=1673 posts) Eye disorder (n=1556 posts) ADHD^a^ (n=1043 posts) Addiction and drug use (n=1001 posts) Allergies (937 n=posts)
r/askdocs	Questions and answers with verified medical professionals	9496 (12.02)	STIs^b^ (n=748 posts) Dermatology; unspecified (n=646 posts) Musculoskeletal conditions (n=598 posts) Allergies (n=465 posts) Anxiety (n=435 posts)
r/amItheasshole	Users post stories to “find out if [they] were wrong”	5473 (6.93)	STIs (n=637 posts) Anxiety (n=461 posts) Abuse (n=400 posts) Nonmedical (n=353 posts) Pregnancy (n=334 posts)
r/relationship_advice	Relationship-based discussions	5322 (6.74)	STIs (n=1190 posts) Abuse (n=530 posts) Dating (n=497 posts) Obsessive-compulsive disorder (n=374 posts) Anxiety (n=358)
r/relationships	Interpersonal relationship–based discussion	2775 (3.51)	STIs (n=420 posts) Abuse (n=390 posts) Obsessive-compulsive disorder (n=281 posts) Dating (n=248 posts) Anxiety (n=222 posts)
r/medical	Medical question–based discussion	2495 (3.16)	Dermatology; unspecified (n=235 posts) STIs (n=175 posts) Musculoskeletal conditions (n=173 posts) Allergies (n=151 posts) Eye disorder (n=114 posts)
r/legaladvice	Forum to ask “simple legal questions”	2227 (2.82)	Addiction and drug use (n=401 posts) Medical billing (n=373 posts) Musculoskeletal conditions (n=110 posts) PTSD^c^ (n=109 posts) STIs (n=102 posts)
r/advice	Advice forum	2134 (2.7)	Obsessive-compulsive disorder (n=186 posts) STIs (n=184 posts) Anxiety (n=168 posts) Addiction and drug use (n=163 posts) ADHD (n=144 posts)
r/teenagers	Intended for users aged 13-19 years to discuss age-appropriate subjects	1974 (2.5)	Dermatology; unspecified (n=591 posts) ADHD (n=154 posts) Eye disorder (n=101 posts) Depression (n=91 posts) Anxiety (n=73 posts)
r/fitness	Physical fitness–based discussion	778 (0.99)	Musculoskeletal conditions (n=152 posts) Dieting (n=77 posts) Bodybuilding (n=69 posts) Gallbladder disorder (n=46 posts) Testosterone use (n=37 posts)
r/science	Forum for new scientific research	489 (0.62)	Addiction and drug use (n=48 posts) HPV^d^ (n=29 posts) Anxiety (n=27 posts) STIs (n=23 posts) Obsessive-compulsive disorder (n=19 posts)
r/diagnoseme	Medical question–based discussion	479 (0.61)	Dermatology; unspecified (n=52 posts) STIs (n=42 posts) Allergies (n=38 posts) Musculoskeletal conditions (n=28 posts) Hypothyroidism (n=25 posts)
r/askdoctorsmeeee	Forum for medical questions	175 (0.22)	Dermatology; unspecified (n=14 posts) Musculoskeletal conditions (n=14 posts) Allergies (n=12 posts) Arrhythmia (n=9 posts) Hypothyroidism (n=9 posts)
r/askdoctors	Forum for medical advice	150 (0.19)	STIs (n=13 posts) Dermatology; unspecified (n=11 posts) Allergies (n=8 posts) Anxiety (n=8 posts) Gallbladder disorder (n=8 posts)
r/medicine	Forum for physicians to discuss medicine	125 (0.16)	Addiction and drug use (n=18 posts) Pregnancy (n=8 posts) Anemia (n=6 posts) Gallbladder disorder (n=5 posts) HIV (n=5 posts)

^a^ADHD: attention-deficit/hyperactivity disorder.

^b^STI: sexually transmitted infection.

^c^PTSD: posttraumatic stress disorder.

^d^HPV: human papillomavirus.

**Table 6:** The top 5 topics by number of posts in the top 15 topic-specific subreddits (describing a certain health-related topic). A description of the subreddit and the total number of posts in the subreddit are provided (N=78,980).

Subreddit	Description	Posts, n (%)	Top 5 topics in each subreddit
r/skincareaddiction	Broad forum to discuss skin	2268 (2.87)	Eye disorder (n=1226 posts) Dermatology; unspecified (n=273 posts) PCOS^a^ (n=225 posts) Allergies (n=87 posts) Eczema (n=73 posts)
r/testosterone	Testosterone replacement therapy and testosterone levels	1746 (2.21)	Vitamins (n=370 posts) Testosterone use (n=264 posts) Hypothyroidism (n=171 posts) Pregnancy (n=105 posts) Anemia (n=103 posts)
r/babybumps	Pregnancy-related discussion	1775 (2.25)	Pregnancy (n=819 posts) Musculoskeletal conditions (n=69 posts) Anxiety (n=58 posts) Menstrual cycle (n=58 posts) STIs^b^ (n=49 posts)
r/ADHD	Forum for people with ADHD^c^ to exchange stories and strategies	1807 (2.29)	ADHD (n=944 posts) Addiction and drug use (n=199 posts) Obsessive-compulsive disorder (n=112 posts) Depression (n=91 posts) Anxiety (n=87 posts)
r/STD	Forum to discuss STDs^d^	1611 (2.04)	STIs (n=1158 posts) HIV (n=75 posts) Anxiety (n=54 posts) HPV^e^ (n=48 posts) Respiratory infection (n=32 posts)
r/Pregnant	Forum to discuss pregnancy	1166 (1.48)	Pregnancy (n=494 posts) Menstrual cycle (n=92 posts) Hematology (n=50 posts) STIs (n=47 posts) Anxiety (n=40 posts)
r/Depression	Support for those struggling with a depressive disorder	1193 (1.51)	Depression (n=255 posts) Obsessive-compulsive disorder (n=236 posts) Anxiety (n=129 posts) Addiction and drug use (n=940 posts) ADHD (n=63 posts)
r/Anxiety	Support for those struggling with anxiety	1215 (1.54)	Anxiety (n=628 posts) Obsessive-compulsive disorder (n=165 posts) Arrhythmia (n=55 posts) PTSD^f^ (n=53 posts) Eye disorder (n=31 posts)
r/birthcontrol	Forum to discuss birth control	1102 (1.4)	Menstrual cycle (n=521 posts) Pregnancy (n=180 posts) STIs (n=70 posts) Anxiety (n=26 posts) Musculoskeletal conditions (n=24 posts)
r/Childfree	Forum for those who choose to not have children	966 (1.22)	Pregnancy (n=580 posts) Menstrual cycle (n=58 posts) Male reproductive health (n=32 posts) Abuse (n=22 posts) Anxiety (n=18 posts)
r/Hypothyroidism	Forum for those with hypothyroidism	779 (0.99)	Hypothyroidism (n=519 posts) Anemia (n=69 posts) Dieting (n=29 posts) Vitamins (n=23 posts) Anxiety (n=19 posts)
r/MentalHealth	Moderated forum to discuss mental health issues	741 (0.94)	Obsessive-compulsive disorder (n=208 posts) Anxiety (n=126 posts) Depression (n=73 posts) ADHD (n=57 posts) Addiction and drug use (n=43 posts)
r/Dentistry	Forum for dental professionals	683 (0.86)	Dentistry (n=418 posts) Dermatology; unspecified (n=28 posts) Allergies (n=24 posts) STIs (n=23 posts) Musculoskeletal conditions (n=19 posts)
r/Diabetes	Forum for those living with diabetes	702 (0.89)	Diabetes (n=491 posts) Eye disorder (n=35 posts) Anemia (n=23 posts) Dieting (n=19 posts) Vitamins (n=15 posts)
r/Infertility	Forum for those dealing with infertility for all reasons	446 (0.56)	Pregnancy (n=110 posts) Menstrual cycle (n=34 posts) Vitamins (n=33 posts) Hypothyroidism (n=29 posts) Endometriosis (n=22 posts)

^a^PCOS: polycystic ovary syndrome.

^b^STI: sexually transmitted infection.

^c^ADHD: attention-deficit/hyperactivity disorder.

^d^STD: sexually transmitted disease.

^e^HPV: human papillomavirus.

^f^PTSD: posttraumatic stress disorder.

### Medical Disciplines Divided by Subreddit

We also analyzed the breakdown of posts in each subreddit differentiated by medical discipline (Figure S1 in Multimedia Appendix 1). The relative frequency of medical disciplines in each subreddit was calculated by dividing the number of posts in a medical discipline by the total number of analyzed posts in that subreddit. Our analysis showed that many subreddits included posts about psychiatry and mental health. This mirrors the breakdown of medical disciplines overall as most posts belonged to that discipline. Some subreddits predominantly contained posts belonging to other medical disciplines. In those cases, the medical discipline often correlated with the subreddit (eg, in *r/hypothyroidism*, most posts belonged to the endocrinology, nutrition, and metabolism medical discipline). However, most subreddits comprised posts from multiple medical disciplines.

## Discussion

### Most Prevalent Medical Topics and Disciplines

By using markers of medical topics identified from broader samples of user data to categorize social media posts into specific diseases, previous studies have been able to predict diagnoses using social media information [13,14,20]. We accomplished a similar goal in our study by identifying topic keywords and using those to assign topics to posts. From there, our analysis revealed which disciplines were most prevalent across Reddit. Similarly to previous studies, we also used keywords to categorize this form of data into medical topics, but in this case, we did this on a broader scale, sorting data into all possible medical disciplines and generally not excluding posts that did not meet predetermined categories.

The 4 medical disciplines with the greatest proportion of posts were psychiatry and mental health; genitourinary and reproductive health; infectious diseases; and endocrinology, nutrition, and metabolism. We note the fact that psychiatry and mental health had approximately twice as many posts as the next highest medical discipline, genitourinary and reproductive health (21,009/79,980, 26.6% vs 10,741/78,980, 13.6% of posts) as another sign that discussion of mental health is common on Reddit.

Psychiatry and mental health, as a discipline, was the most prevalent. In addition to posts about anxiety, this medical discipline comprised posts about depression, addiction, alcoholism, and tobacco use. There were many posts about drug use, drug testing at work, and how drugs would affect other health conditions. Overall, the medical discipline of psychiatry and mental health emphasized the amount of concern that people have about mental health topics and that they were willing to discuss their issues online. The large proportion of posts being about mental health raises the question of whether there is insufficient access to mental health services, whether online discussion of mental health is predominant over other health topics because of anonymity, or both.

Within this discipline, anxiety was another medical topic that frequently appeared, making up 5.43% (4289/78,980) of all posts and being in the top 5 topics in both general and topic-specific subreddits. Interestingly, anxiety was one of the most frequent topics not only in subreddits related to mental health but also in *r/PancreaticCancer*, *r/STD*, and *r/juicing*. Anxiety is a broad topic. Posts in this topic ranged from those about sharing experiences fighting melanoma to anxiety due to marijuana-triggered hallucinations to broad descriptions of “head pressure, headache, jaw pain, throat tension.” Because the description of anxiety is open to interpretation, capturing which posts discuss anxiety is difficult and will result in a variety of posts that mention anxious feelings and worry, but the main subject of the post may not necessarily be anxiety.

We note that the most prevalent medical disciplines and topics can entail feelings of shame and embarrassment, be highly sensitive, or evoke a need for secrecy [33-35]. STIs was the most common topic, making up 7.44% (5876/78,980) of all posts. STIs had a significantly higher relative frequency than the next most common topic, eye disorders. Posts on this topic included many situational descriptions of suspected STIs. An example of an STI post is as follows:

[These] past couple of weeks, my girlfriend (we are in a monogamous relationship) found out that she was having light stringy brown discharge midway through her period cycles...So she went to the doctor and the doctor has prescribed her 1g Azithromycin (one time) and a 1 week course of Metrogyl 400 gms...The doctor also adviced [sic] her that I should take only the Azithromycin as I could have the infection as well (no blood tests or similar were done, the doctor did a physical examination)...

The medical discipline of endocrinology, nutrition, and metabolism, which included posts about hypothyroidism, vitamins, and diabetes, among others, presented an interesting category. Posts discussed checking vitamin D levels and supplements. However, many posts also overlapped with the topic of pregnancy, such as checking vitamin levels during pregnancy and hormone levels. Therefore, it was difficult to parse whether all these posts were truly about endocrinology, nutrition, and metabolism. We also noticed that there were posts mentioning thyroid problems or diseases that were not specific to hypo- or hyperthyroidism. While these posts were more often categorized under hypothyroidism, the endocrine, nutrition, and metabolism medical discipline more accurately captured them under a broader category.

Overall, Reddit holds advantages over other social media platforms because its culture trends toward anonymity. Authors have discussed how the anonymity of communication over the internet reduces social desirability bias and social anxiety. Users are more disinhibited to discuss things openly [5]. Other studies have suggested that the internet is useful for collecting self-reports on risky health behaviors such as alcohol use, drug use, and smoking. Respondents accurately self-reported behaviors online, even ones that were undesirable, because of the perception of privacy [36]. It is established that social media sites with a culture of anonymity allow people to talk about sensitive issues online without fear of social desirability bias. Our study leverages that culture to gather a broader look at all medical disciplines rather than a predetermined set of medical topics. Our methods classified user data into those topics iteratively and broadly into all possible medical disciplines. Therefore, our findings expand on the literature by highlighting the most prevalent disciplines of conversation in a platform-wide study.

Interestingly, the top 5 topics may not be considered top health topics by health care providers. In a recent metric published by Blue Cross Blue Shield (Blue Cross Blue Shield Health Index), the top 10 health topics that had the greatest impact on quality of life in the United States did not include any of the topics that we mentioned previously. Therefore, our study may show that there is a different set of online topics, or it may reinforce that mining internet data reveals a different set of health concerns from those traditionally discussed or considered to be major health issues.

### The Distribution of Topics

The aforementioned skewed distribution of topics suggests to us that conversation on Reddit is concentrated in a minority of discussion topics. This correlates with our supposition that users, when discussing health topics, overwhelmingly post about issues related to mental health, reproductive health, and infectious diseases, as well as endocrine, metabolic, and nutritional issues. The relative paucity of other topics may suggest that these are not discussed either in the general subreddits we focused on or in the topic-specific subreddits in the selection of health-related subreddits provided by Reddit but may be discussed in other subreddits.

As previously noted, 10 topics had no posts assigned to them. This meant that some topics that were created inductively during manual post review did not have any posts assigned to them during automatic topic assignment in the 1-year dataset. This invites us to investigate why certain topics would not have posts assigned to them. One reason is that posts in the subreddits included in the final analysis may not have been assigned to those topics. Another reason is that posts about a certain topic, for example, vaccination, may have also discussed measles, influenza, or human papillomavirus and, subsequently, were assigned to other topics, such as upper respiratory infection and STIs.

In general, users appeared more willing to talk about subjects that would otherwise be uncomfortable to discuss in person. This is in line with other studies that have examined how people are more willing to talk about chronic diseases on social media. They are also willing to share their disease experience in detail. Previous work has found that, on certain disease-oriented subreddits, users freely discuss disease-anatomy relationships, such as cancer-lymph and asthma-lung relationships. The authors write that this openness on subreddits may stem from a desire to find others who share their experience of the disease [24] and users feel reassured by the subreddits’ shared experiences.

### Giving Advice

A subset of subreddits include those where users can post a question with the expectation that a subject matter expert will provide an answer while also being open to public input. On subreddits such as *r/askdocs* and *r/askdoctorsmeeee*, the experts are stated to be health professionals verified by the subreddit moderators. In a way, these accounts are analogous to the quantified influence described in the study by Koo et al [37] and the power accounts in the study by Sugawara et al [38], showing that, similarly to Twitter, medical advice about testing and diagnosis may be heavily influenced by several accounts on Reddit.

*r/askdocs* has approximately 270,000 members, whereas *r/askdoctorsmeeee* currently has approximately 14,400 members. In these subreddits, the most prevalent topics were similar to those found in other subreddits. We believe that many people turn to these forums for advice and that these subreddits are representative of the kinds of conversations that users have elsewhere on the site. However, because of the availability of experts on certain subreddits, Reddit users may be more discerning about the type of advice they expect to receive.

### Limitations

We initially wanted to determine whether there was any correlation between medical topics that were mentioned on Reddit and medical tests that were mentioned on Reddit. However, our review of the Reddit posts demonstrated that users, in general, did not always mention specific medical tests, instead referring or alluding to a medical condition or a broad class of diseases, such as autoimmune diseases or STIs. At times, the content of a post would simply talk about “something being wrong,” making it difficult to classify into a topic.

We noted other limitations, some of which we were able to address. Some posts were included that only had phrases such as “you should get that checked out,” which were too vague to categorize under any topic. However, this problem was mitigated by using posts only from the 95 subreddits, where the frequency of such comments was lower. This was because those select subreddits, being more focused on their specific diseases or topics, had fewer vague comments and tended to have more thoughtful, directed discussion.

It is possible that keyword selection introduced bias into our topic assignments. For example, for the topic of benign prostatic hypertrophy, keywords included “difficulty urinating” and “difficulty peeing,” but these keywords could have also been applicable to a topic such as nephrology (unspecified). However, we mitigated these biases by having multiple reviewers with backgrounds in clinical medicine and correlating our topic assignments with words in UpToDate. This ensured that the keywords we chose would lead to the least overlap between topics.

We also noted that some posts had too much jargon to be easily classifiable into any one topic. In future studies, they should be categorized in the *nonmedical* topic. Some posts, upon manual review, covered multiple health topics. These were the most difficult posts of all. As our algorithm did not have a way of tagging posts with multiple topics, these posts were assigned to only 1 topic. Future refinement of our algorithm could create a tagging system to identify all the major topics in a post, thereby circumventing this issue.

### Future Applications

Our study provides insights into health discussions on Reddit. Our results are generalizable to other social media platforms. Facebook groups and Twitter hashtags allow for topic-based forums where people can find community discussion on health issues similarly to subreddits. Previous studies on Facebook and Twitter topic analyses have shown that social media data can be collected to better understand public sentiment on specific health topics [13-15,19,39,40]. The topic analysis methods we outlined could be applied to other social media platforms, which may show differences in what topics are discussed. Therefore, future platform-wide studies are needed.

We found that topics such as STIs and mental health issues are especially prevalent. These are potentially subjects that patients are not often discussing with their health care providers. As previously discussed, people tend to avoid potentially stigmatizing, embarrassing, and uncomfortable conversation topics, and STIs and mental health often entail such feelings. Therefore, our findings reinforce the need for health care systems to have open discussions about these issues. Health care providers establish a framework for these conversations through currently available questionnaires. The Patient Health Questionnaire–9 is a tool that providers can use to assess for depressive symptoms and mood. Asking for a sexual history during patient visits using the 5 P approach (partners, practices, protection from STIs, past history of STIs, and pregnancy) is a systematic way of starting a conversation about a patient’s sexual history and concerns about STIs and pregnancy, topics that were found to be prevalent in our study. In addition, prior work has shown that, when patients seek health information online, the patient-physician relationship can be improved if the physician asks about this information and discusses it with the patient [41]. Our study supports that physicians and patients should discuss health information that the patient finds online.

Health-related discussions on social media and their effects on health policy remain an active area of research. Broadly, health-related discussions on social media have been cited as a potential impactor on disease surveillance and health care policy, practices, and delivery [42,43]. More recently, the relationship between social media and health policies has been extensively discussed in contexts such as COVID-19 [39,40,44] and mental health [45-49]. Further studies on the impact of social media on health interventions and health outcomes are needed [40]. It has been suggested that social media can play a role in health policy beyond increasing awareness of health issues [50]. Given the volume and wide variety of health topics discussed on Reddit, future health policies may need to address prevalent beliefs promulgated by health-related discussions on social media sites. Guarding against health misinformation remains an important issue on social media and an active area of study [51-53]. Therefore, we support policies that encourage informed use of social media and discourage the spread of misinformation. Governance and education about obtaining health information online is crucial.

### Conclusions

To investigate how people use Reddit to talk about seeking medical advice, we conducted a targeted review of posts that talked about “getting tested” and “checked out.” Using an inductive approach to sort these posts into different medical topics, we found that discussion centered on several medical topics. They were further grouped into medical disciplines, and the infectious disease, reproductive, mental health, and endocrine categories had the greatest prevalence.

Reddit proved to be conducive to gathering information on what people discuss when talking about getting checked or tested. We took a platform-wide approach to this study, aiming to examine posts from a spectrum of subreddits related to health care and medicine. We believe that this approach is unique as it aimed to classify posts into all possible medical disciplines. In addition, based on our review of the literature, this is also one of the few studies conducted on Reddit to understand health discussions. Reddit provides similar advantages to those of other social media platforms in its large user base, wealth of information, and exchange of ideas. However, Reddit is additionally useful for studying communication about health concerns because of several of its features. Subreddits provide structured forums to further sort where discussions are happening on the platform. Several subreddits feature “expert” users, fostering a question-and-answer culture. Most importantly, users may favor Reddit because of its anonymity, which allows them to ask about and discuss potentially embarrassing topics in detail. In this way, Reddit’s unique features prove it to be a viable source of research information that is worth exploring.

This study was a preliminary examination of the discussions occurring on Reddit. Future work on Reddit would likely take a more focused approach. Further exploration would include answering specific questions about certain topics encountered on Reddit and their prevalence in certain subreddits. Ultimately, these findings could be translated into actionable items, such as improving physician-patient interactions and laboratory management practices. Overall, the culture, structure, and wealth of information on Reddit make it a valuable source for continued investigation of health-related discussions.

## Appendix

Multimedia Appendix 1 Analysis of our Reddit dataset.

## Abbreviations

ADHD: attention-deficit/hyperactivity disorder
*ICD-10*: *International Classification of Diseases, 10th Revision*
STI: sexually transmitted infection

## Data Availability

The datasets generated and analyzed during this study are available at the Reddit_public_data repository [54].
