# Supplementary material for: Understanding Health-Related Discussions on Reddit: Development of a Topic Assignment Method and Exploratory Analysis
Source: JMIR Form Res. 2025 Jan 29;9:e55309. doi: 10.2196/55309 (PMC11822319; doi:10.2196/55309)
Supplement: Multimedia Appendix 1 [file formative_v9i1e55309_app1.docx]

**Supplemental Data**

SUPPLEMENTARY TABLE I

EXCLUDED SUBREDDITS FROM FINAL QUERY.

|  | | |
| --- | --- | --- |
| *Games* | -r/darkestdungeon | -r/houseofcards |
| -r/pcgaming | -r/forza | -r/MakingaMurderer |
| -r/apexlegends | -r/godofwar | -r/FlashTV |
| -r/destinythegame | -r/ark | -r/trailerparkboys |
| -r/DotA2 | -r/bioshock | -r/mrrobot |
| -r/starcraft | -r/ed | -r/siliconvalleyhbo |
| -r/smashbros | -r/summonerswar | -r/strangerthings |
| -r/dayz | -r/duellinks | -r/supernatural |
| -r/civ | -r/arma | -r/thegrandtour |
| -r/KerbalSpaceProgram | -r/pathfinderrpg | -r/AmericanHorrorStory |
| -r/masseffect | -r/footballmanagergames | -r/rupaulsdragrace |
| -r/clashofclans | -r/kingdomcome | -r/westworld |
| -r/starbound | -r/subnautica | -r/blackmirror |
| -r/heroesofthestorm | -r/thelastofus | -r/FilthyFrank |
| -r/terraria | -r/lotrmemes | -r/orangeisthenewblack |
| -r/dragonage | -r/otmemes | -r/twinpeaks |
| -r/citiesskylines | -r/doom | -r/bigbrother |
| -r/smite | -r/batman | -r/brooklynninenine |
| -r/bindingofisaac | -r/jrpg | -r/howyoudoin |
| -r/eve | -r/smashbrosultimate | -r/30rock |
| -r/starcitizen | -r/brawlstars | -r/lifeisstrange |
| -r/animalcrossing | -r/anthemthegame | -r/survivor |
| -r/metalgearsolid | -r/mortalkombat | -r/riverdale |
| -r/elitedangerous | -r/sekiro | -r/letterkenny |
| -r/bloodborne | -r/TeamfightTactics | -/r/Pokemon |
| -r/monsterhunter | -r/afkarena | -r/AdventureTime |
| -r/warframe | -r/kingdomhearts | -r/futurama |
| -r/undertale | ***Tech*** | -r/TheLastAirbender |
| -r/thedivision | -r/sysadmin | -r/ArcherFX |
| -r/stardewvalley | -r/engineering | -r/southpark |
| -r/nomansskythegame | -r/compsci | -r/TheSimpsons |
| -r/totalwar | -r/webdev | -r/mylittlepony |
| -r/pathofexile | -r/programmerhumor | -r/rickandmorty |
| -r/ClashRoyale | -r/graphic_design | -r/naruto |
| -r/crusaderkings | -r/mechanicalkeyboards | -r/stevenuniverse |
| -r/dwarffortress | -r/reverseengineering | -r/onepunchman |
| -r/eu4 | -r/itsaunixsystem | -r/BobsBurgers |
| -r/thesims | -r/plex | -r/BoJackHorseman |
| -r/assassinscreed | -r/multicopter | -r/gravityfalls |
| -r/playrust | -r/programminghorror | -r/familyguy |
| -r/forhonor | -r/dailyprogrammer | -r/kingofthehill |
| -r/stellaris | -r/coding | -r/spongebob |
| -r/blackdesertonline | -r/python | -r/dbz |
| -r/factorio | -r/java | -r/DBZDokkanBattle |
| -r/Warhammer | -r/cpp | -r/dragonballfighterz |
| -r/splatoon | -r/security | -r/doctorwho |
| -r/rimworld | -r/buildapc | -r/gallifrey |
| -r/Xcom | -r/buildapcsales | -r/IASIP |
| -r/streetfighter | -r/buildapcforme | -r/the_dennis |
| -r/paydaytheheist | -r/talesfromtechsupport | -r/seinfeld |
| -r/MonsterHunterWorld | -r/techsupportgore | -r/redditwritesseinfeld |
| -r/Seaofthieves | -r/techsupport | -r/seinfeldgifs |
| -r/cyberpunkgame | -r/softwaregore | -r/NetflixBestOf |
| -r/warhammer40k | -r/iiiiiiitttttttttttt | -r/Netflix |
| -r/paladins | ***Shows*** | -r/bestofnetflix |
| -r/osugame | -r/GameOfThrones | -r/scrubs |
| -r/spidermanps4 | -r/BreakingBad | -r/theexpanse |
| -r/persona5 | -r/thewalkingdead | ***Movies*** |
| -r/horizon | -r/community | -r/starwars |
| -r/reddeadredemption | -r/arresteddevelopment | -r/harrypotter |
| -r/reddeadredemption2 | -r/topgear | -r/lotr |
| -r/mountandblade | -r/StarTrek | -r/marvelstudios |
| -r/deadbydaylight | -r/HIMYM | -r/DC_Cinematic |
| -r/farcry | -r/firefly | -r/thanosdidnothingwrong |
| -r/hoi4 | -r/PandR | -r/inthesoulstone |
| -r/warthunder | -r/Sherlock | ***Other*** |
| -r/grandorder | -r/DunderMifflin | -r/cars |
| -r/divinityoriginalsin | -r/BetterCallSaul | -r/nosleep |
| -r/escapefromtarkov | -r/TrueDetective |  |

SUPPLEMENTARY TABLE II

LIST OF TOPICS AND THEIR ICD-10 CATEGORY.

| Topic | ICD-10 category |
| --- | --- |
| STIs | infectious disease |
| eye disorder | ophthalmology |
| anxiety | psychiatry and mental health |
| pregnancy | genitourinary and reproductive health |
| addiction and drug use | psychiatry and mental health |
| musculoskeletal | musculoskeletal and connective tissue |
| adhd | psychiatry and mental health |
| nonmedical | neurology |
| obsessive-compulsive disorder | psychiatry and mental health |
| dermatology_unspecified | dermatology |
| allergies | hematology/immunology |
| hypothyroidism | endocrinology, nutrition, and metabolism |
| abuse | psychiatry and mental health |
| PTSD | psychiatry and mental health |
| menstrual cycle | genitourinary and reproductive health |
| depression | psychiatry and mental health |
| vitamins | endocrinology, nutrition, and metabolism |
| gastroesophageal reflux | gastroenterology |
| anemia | hematology/immunology |
| diabetes | endocrinology, nutrition, and metabolism |
| arrhythmia | cardiovascular |
| dieting | endocrinology, nutrition, and metabolism |
| neurology | genitourinary and reproductive health |
| medical billing | medical, other |
| upper respiratory infection | psychiatry and mental health |
| heart attack | cardiovascular |
| hearing disorder | neurology |
| male reproductive health | genitourinary and reproductive health |
| gallbladder disorder | gastroenterology |
| testosterone use | genitourinary and reproductive health |
| dentistry | dentistry |
| HIV | infectious disease |
| gastrointestinal | gastroenterology |
| urinary tract infection | genitourinary and reproductive health |
| headache | neurology |
| alcoholism | psychiatry and mental health |
| cancer, unspecified | neoplasms |
| HPV | genitourinary and reproductive health |
| PCOS | endocrinology, nutrition, and metabolism |
| allergic reaction | hematology/immunology |
| breast cancer | neoplasms |
| miscarriage | genitourinary and reproductive health |
| female reproductive health | ophthalmology |
| eczema | dermatology |
| hematology | cardiovascular |
| cardiology, unspecified | cardiovascular |
| endometriosis | genitourinary and reproductive health |
| autism | psychiatry and mental health |
| benign prostatic hypertrophy | genitourinary and reproductive health |
| respiratory unspecified | pulmonology |
| psychiatry | psychiatry and mental health |
| influenza | infectious disease |
| nephrology | musculoskeletal and connective tissue |
| bodybuilding | endocrinology, nutrition, and metabolism |
| tobacco use | psychiatry and mental health |
| LGBT health | psychiatry and mental health |
| colon cancer | neoplasms |
| celiac | gastroenterology |
| obesity | endocrinology, nutrition, and metabolism |
| lupus | hematology/immunology |
| hip fracture | musculoskeletal and connective tissue |
| genetic testing | medical, other |
| hypercholesterolemia | cardiovascular |
| rheumatology | musculoskeletal and connective tissue |
| measles | infectious disease |
| hepatitis | gastroenterology |
| pneumonia | pulmonology |
| stomach ulcer | gastroenterology |
| multiple sclerosis | neurology |
| ear nose throat | otolaryngology |
| hyperthyroidism | endocrinology, nutrition, and metabolism |
| endocrinology | endocrinology, nutrition, and metabolism |
| asthma | pulmonology |
| body dysmorphic disorder | psychiatry and mental health |
| cystic fibrosis | pulmonology |
| dementia | psychiatry and mental health |
| gestational diabetes | genitourinary and reproductive health |
| infectious mononucleosis | infectious disease |
| Lyme | infectious disease |
| testicular torsion | genitourinary and reproductive health |
| thyroid, unspecified | endocrinology, nutrition, and metabolism |
| vaccination | infectious disease |

SUPPLEMENTARY TABLE III

SUBREDDITS ADDITIONALLY EXCLUDED IN STEP 3.

| ACL | eagles | pkmntcgtrades |
| --- | --- | --- |
| Albuquerque | Fantasyfootball | pokemongospoofing |
| amd | Ford | politicalhumor |
| applehelp | freefolk | politics |
| artc | gaming | rit |
| askeurope | gearsofwar | rupaulsdragrace |
| askmrp | Genealogy | Sat |
| assassinscreed | FGnv | selfharm |
| babybumps | Golfgti | shadownet |
| battlefield_live | gout | Sneakers |
| birthcontrol | greenbaypackers | sousvide |
| bitcoin | gtaonline | step1 |
| bouldering | Guitar | summonerswar |
| breastfeeding | inceltears | svexchange |
| c25k | indianmotorcycle | sysadmin |
| cars | infertility | talesfromthefrontdesk |
| cats | kappa | teenmomunfiltered |
| childfree | kidneydisease | teslamotors |
| churning | leagueoflegends | testosterone |
| churningcanada | livestreamfail | tf2 |
| citiesskylines | Lyme | thriftstorehauls |
| comedyheaven | mechanicadvice | ukpersonalfinance |
| cosmetology | MMA | warframe |
| creditcards | mtvchallenge | watches |
| CysticFibrosis | nhl | weddingplanners |
| Dallas | nosleep | woohah |
| dccomics | opiates | worldnews |
| destinythegame | patriots |  |
| dreamcast | pcgaming |  |
| dxm | pitbulls |  |


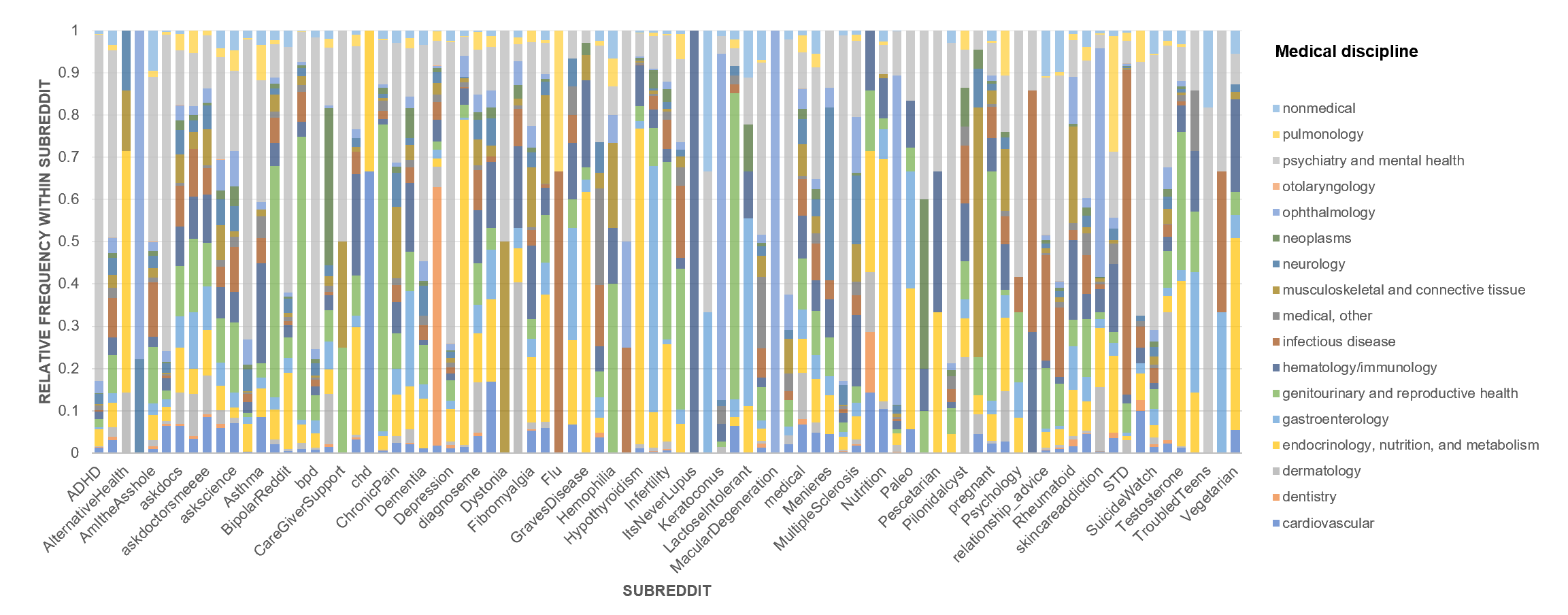


**Supplemental Figure 1. Frequency of medical disciplines within each subreddit.** The frequency of each medical discipline within a given subreddit is represented. Subreddits shown are those designated as relevant in our study. Medical discipline are the same as those detailed in the main text.
